# Regional brain dysregulation of Ca^2+^-handling systems in ketamine-induced rat model of experimental psychosis

**DOI:** 10.1007/s00441-015-2332-3

**Published:** 2015-12-21

**Authors:** Malwina Lisek, Tomasz Boczek, Bozena Ferenc, Ludmila Zylinska

**Affiliations:** Department of Molecular Neurochemistry, Faculty of Health Sciences, Medical University of Lodz, 6/8 Mazowiecka Street, 92215 Lodz, Poland

**Keywords:** Calcium homeostasis, Ketamine, NMDA receptor, Experimental psychosis, Rat

## Abstract

Chronic N-methyl-D-aspartate receptor (NMDAR) antagonist treatment can provide valuable neurochemical and neuroanatomical models of experimental psychosis. One such antagonist, ketamine, with its short half-time and well-documented psychotomimetic action, has cognitive effects resembling various aspects of schizophrenia-like symptoms. In order to obtain insights into possible relationships between Ca^2+^ homeostasis and schizophrenia-related symptoms, we investigate the effects of chronic ketamine administration on intracellular Ca^2+^ levels in various brain regions and on the expression level of key members of the neuronal Ca^2+^-handling system in rats. We show increased intracellular [Ca^2+^] in all of the examined brain regions following ketamine treatment but an altered cytosolic Ca^2+^ level correlated with hyperlocomotor activity was only established for the cortex and striatum. Our findings also suggest that an imbalance in the expression between the calcium “on” and “off” systems contributes to the deregulation of brain Ca^2+^ homeostasis in our ketamine-induced model of experimental psychosis. Identification of the genes whose expression is affected by ketamine treatment indicates their involvement as putative etiological factors in schizophrenia.

## Introduction

Although the pathophysiology of schizophrenia has not yet been elucidated, clinical findings have led to the hypothesis that the hypofunction of N-methyl-D-aspartate receptors (NMDAR) underlies the induction of psychotic symptoms (Lajtha et al. 2009). This so-called “glutamate hypothesis” has developed because NMDAR non-competitive antagonists, such as ketamine, produce schizophrenia-like symptoms in healthy humans (Krystal et al. 1994). In rodents, the administration of NMDAR blockers produces analogous behavioral abnormalities including increased locomotion, ataxia, cognitive defects and social withdrawal (Lajtha et al. 2009). Despite mimicking a spectrum of psychotic symptoms, the simple blockade of NMDAR cannot explain all accompanying defects, which are likely to have a multifactorial origin. The idea proposed by Jimerson et al. in 1979 (Jimerson et al. 1979) and supported by further findings (Lidow 2003) that altered intracellular Ca^2+^ signaling contributes to schizophrenia etiology and has now returned to the mainstream of schizophrenia research. According to this theory, disturbed Ca^2+^ homeostasis and subsequent dysfunctions in Ca^2+^-regulated signal transduction cause schizophrenic symptomology. Indeed, postmortem studies have consistently reported structural and functional abnormalities in the prefrontal cortex, hippocampus and cerebellum and dysregulation of the striatal dopaminergic system in schizophrenia (Lajtha et al. 2009).

Calcium homeostasis in neural cells is maintained by items in the Ca^2+^ signaling toolkit called “on” and “off” mechanisms. The activation of “on” mechanisms increases the intracellular Ca^2+^ concentration ([Ca^2+^]_c_), whereas “off” systems tend to restore the resting Ca^2+^ level. Most of the extracellular Ca^2+^ enters the cell through voltage-dependent Ca^2+^ channels and store-operated Ca^2+^ channels (SOCs). Voltage-dependent Ca^2+^ channels encoded by the CACNA1A-S genes are key transducers of electrical excitability to intracellular Ca^2+^ transients, which, in turn, initiate neurotransmission by triggering vesicular release (Stanley 1993). SOCs respond to endoplasmic reticulum (ER) store depletion and, together with STIM1 and ORAI1 proteins, participate in ER refilling via a store-operated Ca^2+^ entry mechanism. Moreover, a few members of canonical transient receptor potential channels (TRPC), which are nonselective plasma membrane cation channels with a preference for Ca^2+^ over Na^+^, are considered to be SOCs (Smyth et al. 2010).

Ca^2+^ clearance mechanisms aimed at restoring the basal Ca^2+^ level during the “off” phase include a network of plasma-membrane-located Na^+^/Ca^2+^ exchangers (NCX1-3), ATP-dependent Ca^2+^ pumps (PMCA1-4), sarco(endo)plasmic reticulum Ca^2+^-ATPases (neuronal SERCA2-3) and secretory-pathway Ca^2+^-ATPases (SPCA1-2) located in the membrane of the Golgi apparatus. Furthermore, significant amounts of cytosolic Ca^2+^ can also be accumulated by mitochondria. This Ca^2+^ uptake undertaken by the mitochondrial calcium uniporter (MCU) shapes spatiotemporal Ca^2+^ signals and might trigger cell death (Kirichok et al. 2004).

However, more recent studies have shown that chronic NMDAR antagonist treatment can provide a valuable neurochemical and neuroanatomical model of experimental psychosis (Lajtha et al. 2009). Ketamine, because of its short half-time and well-documented psychotomimetic action, has cognitive effects resembling various aspects of schizophrenia-like symptoms (Stefani and Moghaddam 2005; Neilla et al. 2010). Therefore, in order to obtain insights into a possible relationship between Ca^2+^ homeostasis and schizophrenia-related symptoms, we investigate the effects of chronic ketamine administration on the expression level of key players in the neuronal Ca^2+^-handling system. Our findings suggest that imbalanced expression between calcium “on” and “off” systems contributes to the deregulation of brain Ca^2+^ homeostasis in a ketamine-induced model of experimental psychosis.

## Materials and methods

### Reagents

All reagents, if not separately mentioned, were purchased from Sigma-Aldrich (Germany). The 5×HOT FIREPol EvaGreen qPCR Mix was from Solis BioDyne (Lithuania). The M-MLV reverse transcriptase, Trizol reagent and Fluo-4 Direct Calcium Assay Kit were from Life Technologies (USA). We obtained the CytoTox-ONE assay from Promega (USA). The ATPLite 1 step Luminescence Assay System was purchased in Perkin-Elmer (USA). Primers were synthesized in the Institute of Biochemistry and Biophysics (Poland).

### Animals and drug treatment

The 10– to 12-week-old male Wistar rats (in-house animal facility, Medical University of Lodz) were group-housed in laboratory cages and kept under a controlled temperature (23 ± 2 °C) with a 12-h light/dark cycle and with food and water provided ad libitum. For 5 consecutive days, randomly chosen rats (*n* = 12) were injected intraperitoneally with ketamine (30 mg/kg) dissolved in physiological saline. Injections of ketamine for 5 consecutive days were shown to induce the behavioral and molecular changes related to schizophrenia-like alterations (Becker et al. 2003). The control group (*n* = 12) was injected with saline only (0.5 ml/kg). All animal experimentation was conducted in accordance with Directive 2010/63/EU of the European Parliament on the protection of animals used for scientific purposes. All the procedures used were also approved by the Institutional Animal Care and Use Committee at Medical University of Lodz.

### Behavioral testing

On day 5 and approximately 2 h after final injection, animals were placed in the open field (100 cm × 100 cm × 40 cm) and were allowed to explore the arena freely for 10 min. Then, locomotor activity was continuously measured over 20 min by using a webcam connected to the ANY-maze video tracking system (Stoelting, USA). The stereotypic behavior was evaluated by counting the amount of turning, weaving and bobbing. Weaving and head bobbing were assessed by counting the neck wave up and down and left and right. Turning was measured by counting the number of turns around. The cumulative stereotypy was determined for each animal and was expressed as the sum of each of the scores over 20 min. Behavioral tests were performed between 10 a.m. and 3 p.m.

### Brain sample preparation

Immediately after behavioral testing and no longer than 3 h following final ketamine administration, animals were decapitated by using a guillotine. Brains were freshly dissected and used immediately or were frozen for further use. For Ca^2+^ measurements, brains were processed essentially as described in Rothman (1984). In brief, pieces of the cortex, hippocampus, cerebellum and striatum (∼200 mg each) were dissociated in 0.1 % trypsin in buffer A (10 mM TRIS–HCl, 138 mM NaCl, 5 mM KCl, 4 mM NaHCO_3_, 0.3 mM Na_2_HPO_4_, 0.44 mM KH_2_PO_4_, 5.6 mM glucose, 0.5 mM phenylmethane sulfonylfluoride, pH 7.4). Following a 15-min incubation at 37 °C, samples were suspended in 1 ml trypsin-free buffer A and passed through a Pasteur pipette with a flame-narrowed tip. Then, cells from particular areas were centrifuged twice at 200*g* for 5 min. The pellets were suspended in buffer B (buffer A supplemented with 5 mM CaCl_2_, 1 mM MgCl_2_) and used for experiments.

### Viability assays

Following dissociation, cellular viability was assessed by means of two independent assays. The CytoTox-ONE assay was used to measure lactate dehydrogenase (LDH) released into the extracellular medium. For this purpose, 100 μl cell suspension in buffer B (for composition, see Brain sample preparation) was taken from the control and ketamine-treated groups after 2, 4, 8 and 12 h of incubation and was placed into 96-well plates. LDH activity was determined according to the manufacturer’s protocol. Cells lysed with 0.1 % Triton X-100 were used to estimate the maximal LDH amount available for release (0 % viability). A second method was applied to quantify the intracellular ATP content by using the ATPLite 1 Step Luminescence Assay System. Cells were directly plated into 96-well plates and the luminescent signal was recorded at various time intervals (2, 4, 8 and 12 h following plating) according to the manual provided by the supplier. Luminescence detected by a GloMax 20/20 luminometer (Promega) was converted to ATP values based on a calibration curve and was expressed as nanomoles per milligram of protein. The protein concentration was quantified by using Bio-Rad Protein Assay.

### Calcium measurement

Cell suspension (100 μl) obtained as in Brain sample preparation was immediately transferred to 96-well plates. An equal volume of 2× Fluo-4 Direct calcium reagent loading solution was added to each well and plates were incubated at 37 °C for 1 h. Fluorescence was measured by using a Victor X3 plate reader (Perkin-Elmer) set for excitation at 488 nm and emission at 535 nm. The signal was calibrated by the addition of 10 μM ionomycin to obtain Fmax and 10 mM EGTA to record Fmin. Changes in Fluo-4 fluorescence were converted to absolute [Ca^2+^]_c_ according to the equation [Ca^2+^]_free_ = K_d_ ([F-Fmin]/[Fmax-F]), where K_d_ = 345 nM.

### Gene expression

Total RNA from dissected brain regions such as the cortex, cerebellum, hippocampus and striatum was extracted with Trizol Reagent following the manufacturer’s instructions. Single-stranded cDNA was synthesized from 1 μg total RNA with oligo(dT) primers in a 20-μl mixture by using M-MLV Reverse Transcriptase. Real-time quantitative polymerase chain reaction (qPCR) was carried out with the Abi Prism 7000 sequence detection system (Applied Biosystems) by using the EvaGreen qPCR Mix and results were analyzed with accompanying software. Amplifications were generated over 15 min at 95 °C followed by 40 cycles at 95 °C for 15 s, 60 °C for 30 s and 72 °C for 30 s. The same conditions were used for all analyzed genes. A melting curve was performed to assess the specificity of the primers listed in Table 1. Relative quantification of gene expression was performed with the 2^-ΔΔCt^ method (Livak and Schmittgen 2001) by using the expression of the *Gapdh* (*D-glyceraldehyde-3-phosphate dehydrogenase*) housekeeping gene to normalize the data.

**Table 1:** Primers used in real-time polymerase chain reaction for amplification of target genes

Gene	Primer sequences	°C	NCBI number
PMCA1	F: 5’-CCTGAGGTACCAGAGGCAATAAA-3’	57,71	NM_053311.1
	R: 5’-TGGGTGTAAAATACCGCATTTG-3’	57,51	
PMCA2	F: 5’-ACCGTGGTGCAGGCCTATGT-3’	56,76	NM_012508.5
	R: 5’-GGCAATGGCGTTGACCAGCA-3’	57,95	
PMCA3	F: 5’-AGGCCTGGCAGACAACACCA-3’	57,58	NM_133288.1
	R: 5’-TCCCACACCAGCTGCAGGAA-3’	58,06	
PMCA4	F: 5’-ACGCGGTGTATCAGCTCGGA-3’	58,29	NM_0010058
	R: 5’-AGTGCTGGCTGGGTGGTGAA-3’	57,64	71.1
SERCA2	F: 5’-TCTGTCATTCGGGAGTGGGG-3’	55,9	NM_001110139.2
	R: 5’-GCCCACACAGCCAACGAAAG-3’	55,9	
SERCA3	F: 5’-CCACCAGGGACACACCCCCA-3’	60,0	NM_012914.1
	R: 5’-AATGCCCGCCCGAGAACAGC-3’	57,9	
SPCA1	F: 5’-CCAGTGTGGCCGTGGCTGAC-3’	60,0	NM_131907.2
	R: 5’-TCAGCCTGGAGAAGGCCTGCAA-3’	58,6	
SPCA2	F: 5’-CCCTTCGCCACTGTATCCAAT-3’	54,4	NM_001291454.1
	R: 5’-CTCTCGGTTGCTGTAACGTCAT-3’	54,8	
NCX1	F: 5’-CCCAAGCTTAATGGAGAGACCACCAAGAC-3’	62,9	NM_001270779.1
	R: 5’-CGCGGATCCTTGGAAGCTGGTCTGTCTCC-3’	67,2	
NCX2	F: 5’-CCCAAGCTTCAGACTGCAAGGAGGGTGTC-3’	65,8	NM_078619.1
	R: 5’-CGCGGATCCAATCACCAGCAATGAACCCG-3’	68,7	
NCX3	F: 5’-CCCAAGCTTCAGACTGCAAGGAGGGTGTC-3’	65,7	NM_078620.2
	R: 5’-CGCGGATCCAATCACCAGCAATGAACCCG-3’	65,7	
TRPC1	F: 5’-CACAGTGGGCTTGGCCGGAG-3’	60,0	NM_053558.1
	R: 5’-CCGCAAGCACGAGGCCAGTT-3’	57,9	
TRPC3	F: 5’-GGCACAAGGCGTCGCTGAGT-3’	57,9	NM_021771.2
	R: 5’-GAAGGCCCAAGGCCACGACC-3’	60,0	
TRPC6	F: 5’-GGGGACCTCGCTCGTCCGAA-3’	60,0	NM_053559.1
	R: 5’-CTGCCATGGTCTGCTGCCGT-3’	57,9	
CACNA1a	F: 5’-GCCCGGAGCGCAGAGGATGTA-3’	60,2	NM_012918.3
	R: 5’-TGGCGGACAGGGATGGGGTT-3’	57,9	
CACNA1b	F: 5’-CAGCCCCATGTCTGCTGCCAA-3’	58,3	NM_147141.1
	R: 5’-GCCGAGTTCTGCTGCGGTGA-3’	57,9	
CACNA1c	F: 5’-GCATCTCCATCACCTTCTTC-3’	51,8	NM_012517.2
	R: 5’-CAAATACCTGCATCCCAATC-3’	49,7	
CACNA1d	F: 5’-CTACAGGCGGGATTAAGGAC-3’	53,8	NM_017298.1
	R: 5’-GTATTGGTCTGCTGAAGGGA-3’	51,8	
CACNA1h	F: 5’-CAGCGCCGGTGAGAGCTTCC-3’	60,0	NM_153814.1
	R: 5’-TGGGACCGGCTGTTCCTCGT-3’	57,9	
STIM1	F: 5’-TGCGCTCGTCTTGCCCTGTG-3’	57,9	NM_001108496.2
	R: 5’-TGCGGACGGCCTCAAAGCTG-3’	57,9	
ORAI1	F: 5’-CACCGTCATCGGGACGCTGC-3’	60,0	NM_001013982.1
	R: 5’-CCGGTTCGGTGGGTGGCTTG-3’	60,0	
MCU	F: 5’-AGTACGGTTGTCCCTCTGATG-3’	56,7	NM_001106398.1
	R: 5’-AGTGGTCCTCTTCTCCGCTTTC-3’	56,7	
GAPDH	F: 5’-GGTTACCAGGGCTGCCTTCT-3’	55,9	NG_028301.1
	R: 5’-CTTCCCATTCTCAGCCTTGACT-3”	54,8	

### Statistical analysis

The data are shown as means ± SEM. The number of animals used in particular experiments is given below each figure. The results were analyzed by using STATISTICA 8.0 (StatSoft). The two-tailed unpaired Student’s t-test (or the Mann–Whitney U Test) and one-way analysis of variance (or Kruskal-Wallis test depending on the normality of data) were used when appropriate. *P* < 0.05 was considered as statistically significant. Correlations were assessed by using Pearson’s correlations and were made by using GraphPad Prism software version 5.

## Results

### Animal behavior

**Fig. 1:**
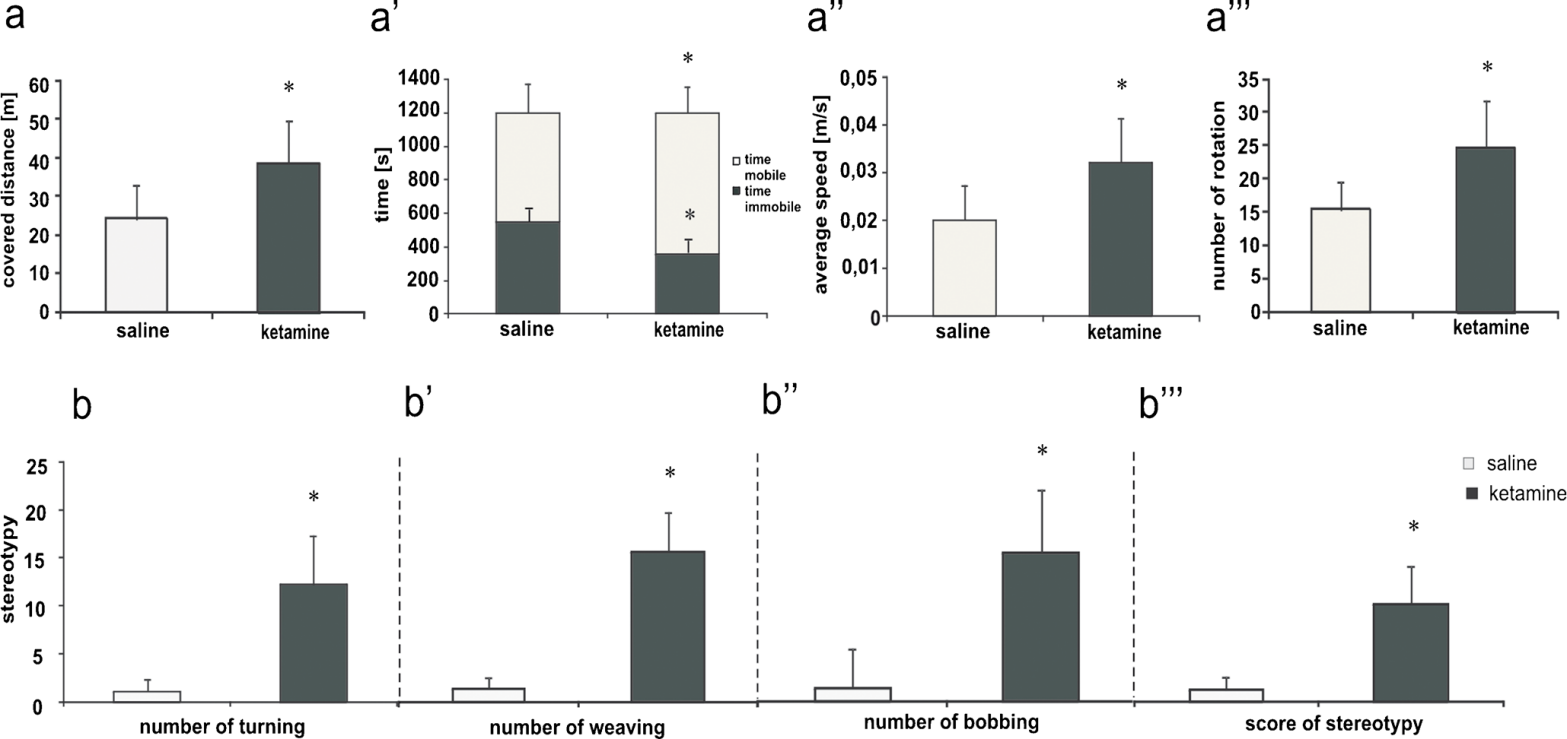
Behavioral evaluation of saline and ketamine injected rats. **a-a’’’** Locomotor activity. **b-b’’’** Stereotypy. Animal behavior was scored by the ANY-maze video tracking system. Tests were started 2 h after the last ketaamine injection and rats were continuously monitored over 20 min. Statistical differences between saline and ketamine-treated rats are indicated by *P < 0.05; control group *n* = 12, tested group *n* = 12

Administration of subanesthetic doses of ketamine (30 mg/kg) promoted behavioral alterations significantly different from those observed in the saline-treated group. Ketamine-injected rats exhibited continuous locomotion with a quick gait. Statistical analysis revealed considerably higher locomotor activity in this group, as reflected by the significant increase in the total distance travelled (*P* = 0.02; Fig. 1a), mobility time (*P* = 0.016; Fig. 1a’), average speed (*P* = 0.019; Fig. 1a’’) and number of body rotations (*P* = 0.013; Fig. 1a’’’). In addition, the use of ketamine produced severe stereotyped movements. As shown in Fig. 1b, cumulative turning (*P* = 0.042; Fig. 1b), weaving (*P* = 0.041; Fig. 1b’), head bobbing counts (*P* = 0.024; Fig. 1b’’) and cumulative scored stereotypy (*P* = 0.038; Fig. 1b’’’) were significantly higher in drug-administered rats.

### Effect of ketamine on cell viability

**Fig. 2:**
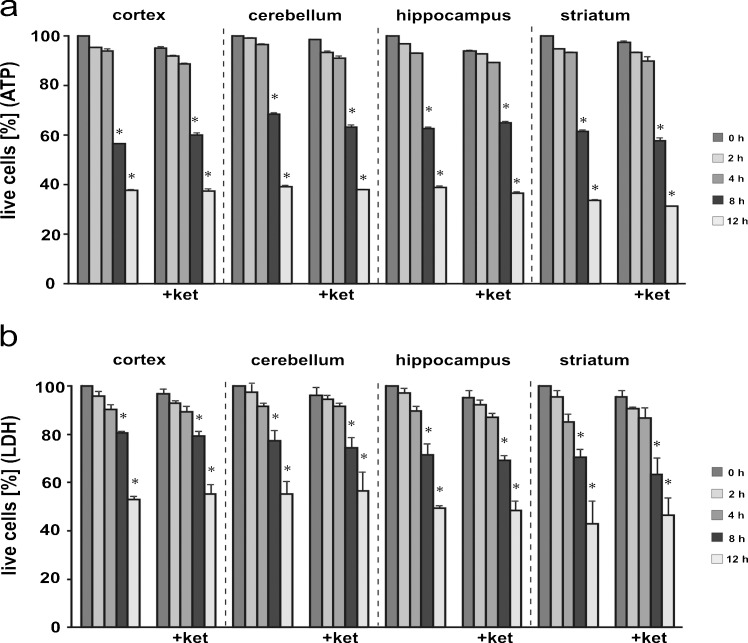
Viability of isolated brain cells. The time course of cell death was assessed by determination of the intracellular ATP level (**a**) and lactate dehydrogenase (*LDH*) activity (**b**). The ATP level at time 0 was taken as 100 % of live cells. For the LDH assay, % cytoxicity was calculated by using the formula (experimental LDH activity)/(maximum LDH activity). Maximum LDH release was achieved by lysing cells with 0.1 % Triton X-100. Statistical differences vs. time 0 are indicated by *P < 0.05. control group (*left*) *n* = 7; ketamine-treated group (*+ket*) *n* = 7

To avoid a potential influence of cell death on the analyzed biochemical changes, we first monitored the mortality of the cells isolated from brain areas by measuring the intracellular ATP level and LDH release (Reifert et al. 2011). ATP content decreased in a time-dependent manner, which became statistically significant in all analyzed brain regions after 8 h (Fig. 2a). To confirm, we next measured the release of LDH that reflected the compromised membrane integrity. The profile of kinetics and the extent of LDH release were similar to the ATP loss (Fig. 2b). This indicates that the dissociation procedure used here did not disturb plasma membrane integrity, which remained intact for at least 4 h.

### Effect of ketamine on Ca^2+^ homeostasis

**Fig. 3:**
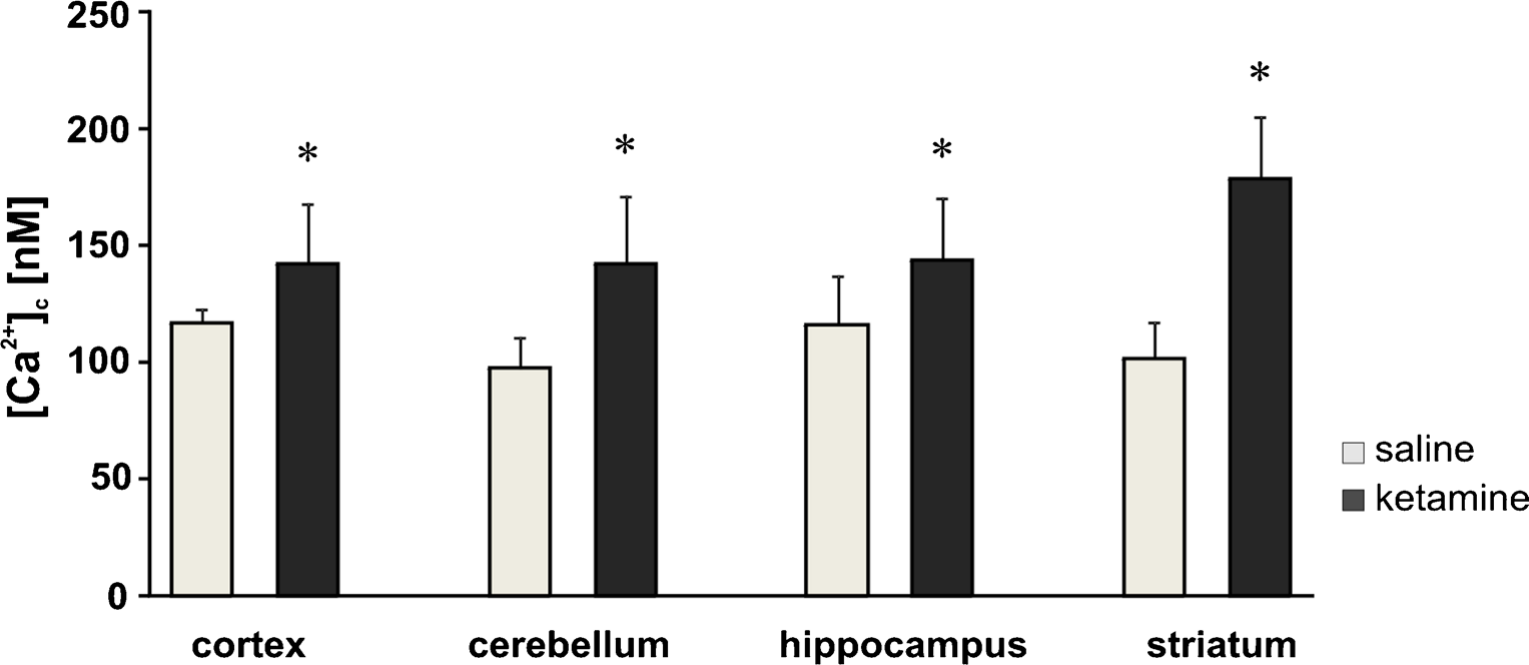
Measurements of intracellular Ca^2+^ (*[Ca* ^*2+*^ *]*_*c*_) in isolated brain cells. The resting calcium level in ketamine-treated or saline-treated brain cells was determined fluorometrically by using the Fluo-4 calcium indicator. Statistical differences, tested by the Mann–Whitney U test, between saline-treated (*n* = 12) and ketamine-treated (*n* = 12) rats are indicated by *P < 0.05

**Fig. 4:**
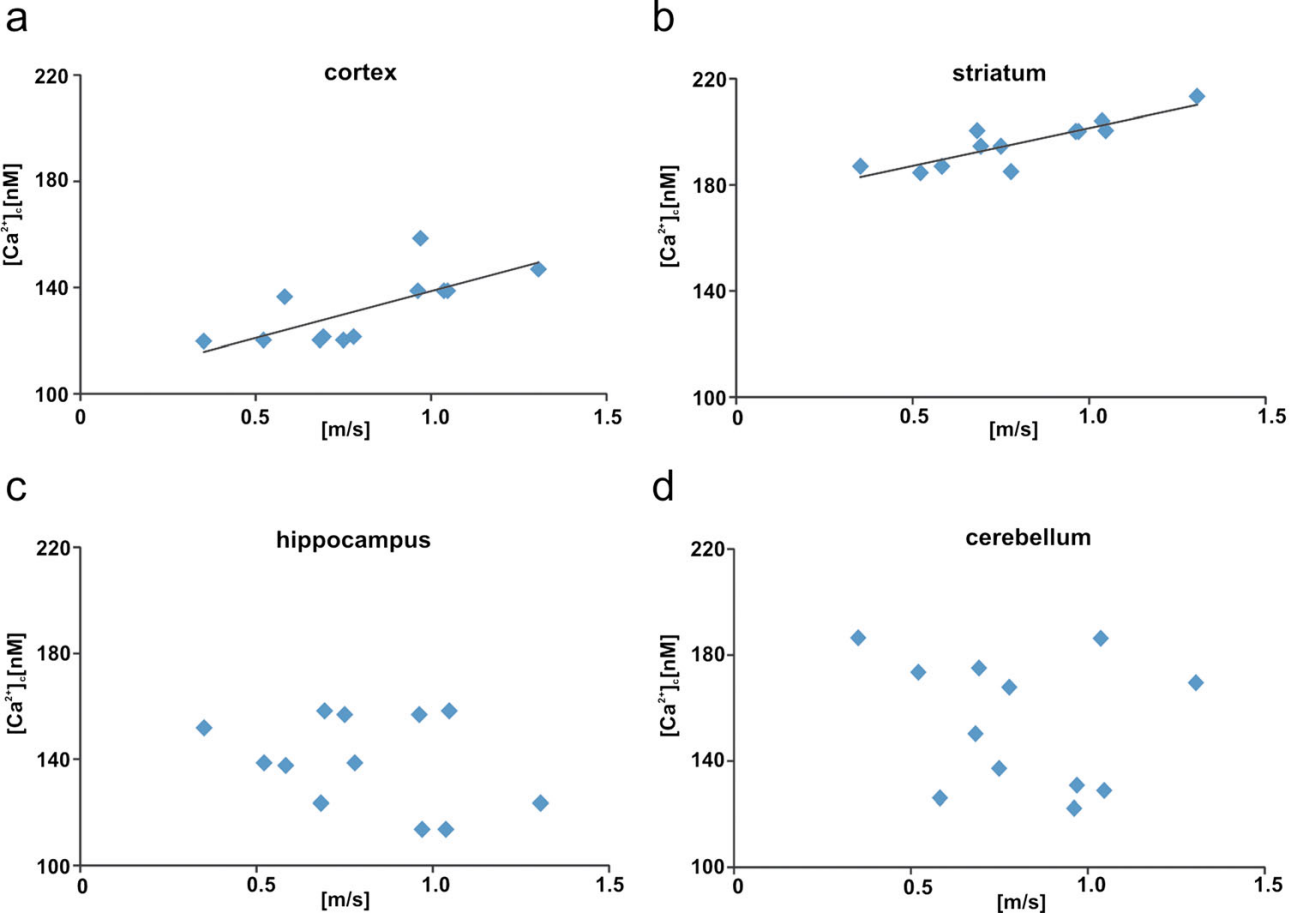
Correlations between resting [Ca^2+^]_c_ and locomotor activity (average speed) in ketamine-injected rats. The *line* on the graphs represents the regressive line. Correlations assessed for selected brain areas: cortex (*r* = 0.705, *P* = 0.01, **a**), striatum (*r* = 0.864, *P* = 0.001, **b**), hippocampus (*r* = −0.33, *P* = 0.29, **c**), cerebellum (*r* = −0.21, *P* = 0.5, **d**). *n* = 12

Within 4 h after isolation of cells from brain areas, we measured [Ca^2+^]_c_ with the Fluo-4 calcium indicator. Brain-region-dependent elevations of [Ca^2+^]_c_ in the ketamine-injected group were detected in comparison with control rats (Fig. 3). In the ketamine-treated rats, a definite increase by ∼80 nM (*P* = 0.0001) was observed in cells isolated from the striatum, followed by a change in cerebellar cells by ∼40 nM (*P* = 0.004). The resting [Ca^2+^]_c_ in the cortical and hippocampal cells was higher by ∼20 nM (*P* = 0.023, *P* = 0.042, respectively). In addition, a positive correlation was found between the locomotor activity in the rats and the increased calcium level in the cortex cells (*r* = 0.705, *P* = 0.01; Fig. 4a) and the striatum cells (*r* = 0.864, *P* = 0.001; Fig. 4b) but not in the hippocampus cells (*r* = −0.33, *P* = 0.29; Fig. 4c) or in the cerebellum cells (*r* = −0.21, *P* = 0.5; Fig. 4d).

### Analysis of gene expression in ketamine-treated rats

Since [Ca^2+^]_c_ was altered in ketamine-treated rats, we next analyzed the transcripts levels of selected elements of Ca^2+^-handling systems, including: voltage-dependent Ca^2+^ channels (CACNA1a, CACNA1b, CACNA1c, CACNA1d, CACNA1h), calcium pumps (PMCA1, PMCA2, PMCA3, PMCA4, SERCA2, SERCA3, SPCA1, SPCA2), exchangers (NCX1, NCX2, NCX3), participants in the store-operated Ca^2+^ entry mechanism (TRPC1, TRPC3, TRPC6, ORAI1, STIM1) and the MCU.

#### Cortex

**Fig. 5:**
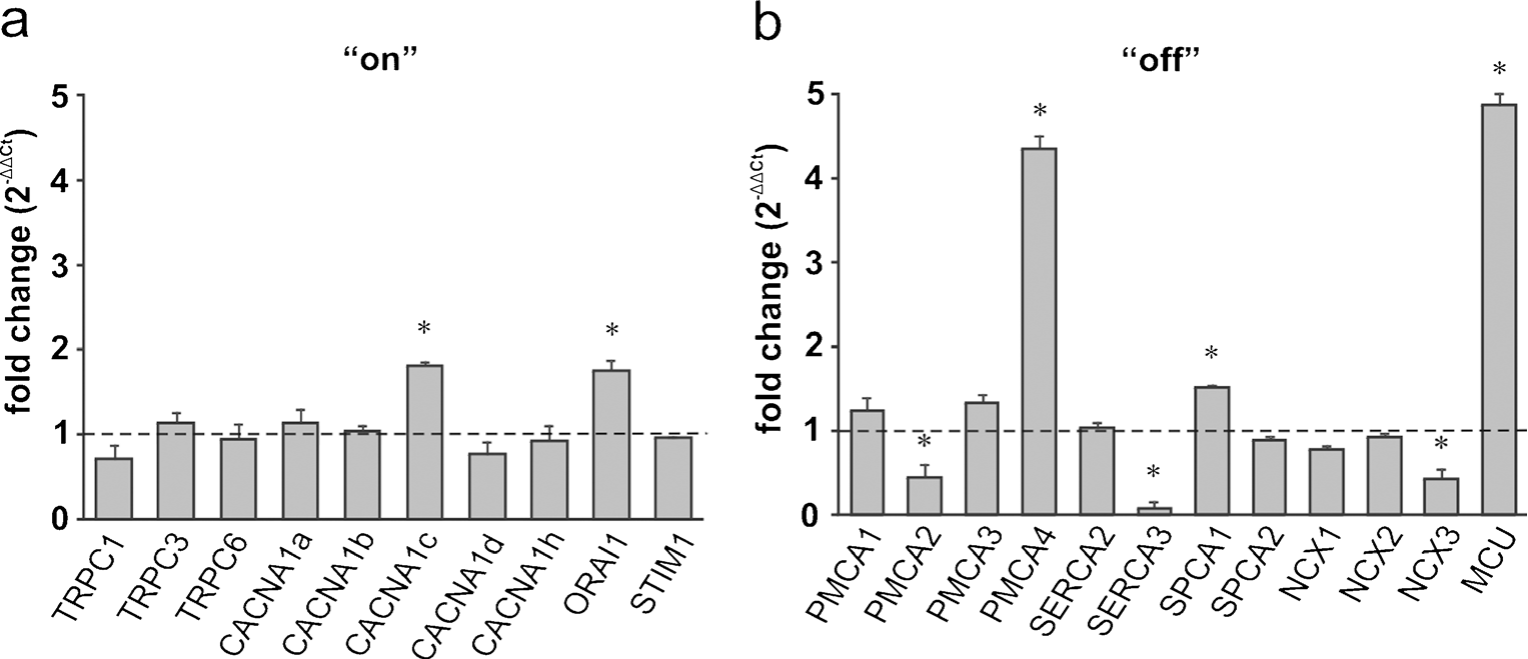
Ketamine-induced changes in the expression of the participants in the Ca^2+^-handling system in the cortex. The gene expression level for the “on” (**a**) and “off” (**b**) systems was assessed by the real-time polymerase chain reaction (PCR). The results are presented as relative units obtained after normalization to the D-glyceraldehyde-3-phosphate dehydrogenase (Gapdh) reference gene. The expression level of each gene in control samples (saline-injected rats) was taken as 1 (*dotted line*). Statistical differences between saline-treated and ketamine-treated rats are indicated, *P < 0.05. *n* = 12

Among the calcium “on” mechanisms, a pronounced increase in the expression level was detected for CACNA1c (*P* = 0.042) and ORAI1 (*P* = 0.047; Fig. 5a). At the same time, the expression of another member of the store-operated Ca^2+^ entry mechanism, namely TRPC1, was decreased (*P* = 0.040). Within the calcium removal systems, the greatest up-regulation in response to ketamine was seen for MCU (*P* = 0.029), PMCA4 (*P* = 0.0019) and SPCA1 (*P* = 0.025), whereas the expression of PMCA2 (*P* = 0.041) and SERCA3 (*P* = 0.045) was reduced (Fig. 5b). Additionally, the down-regulation of the NCX3 isoform was observed (*P* = 0.017).

#### Cerebellum

**Fig. 6:**
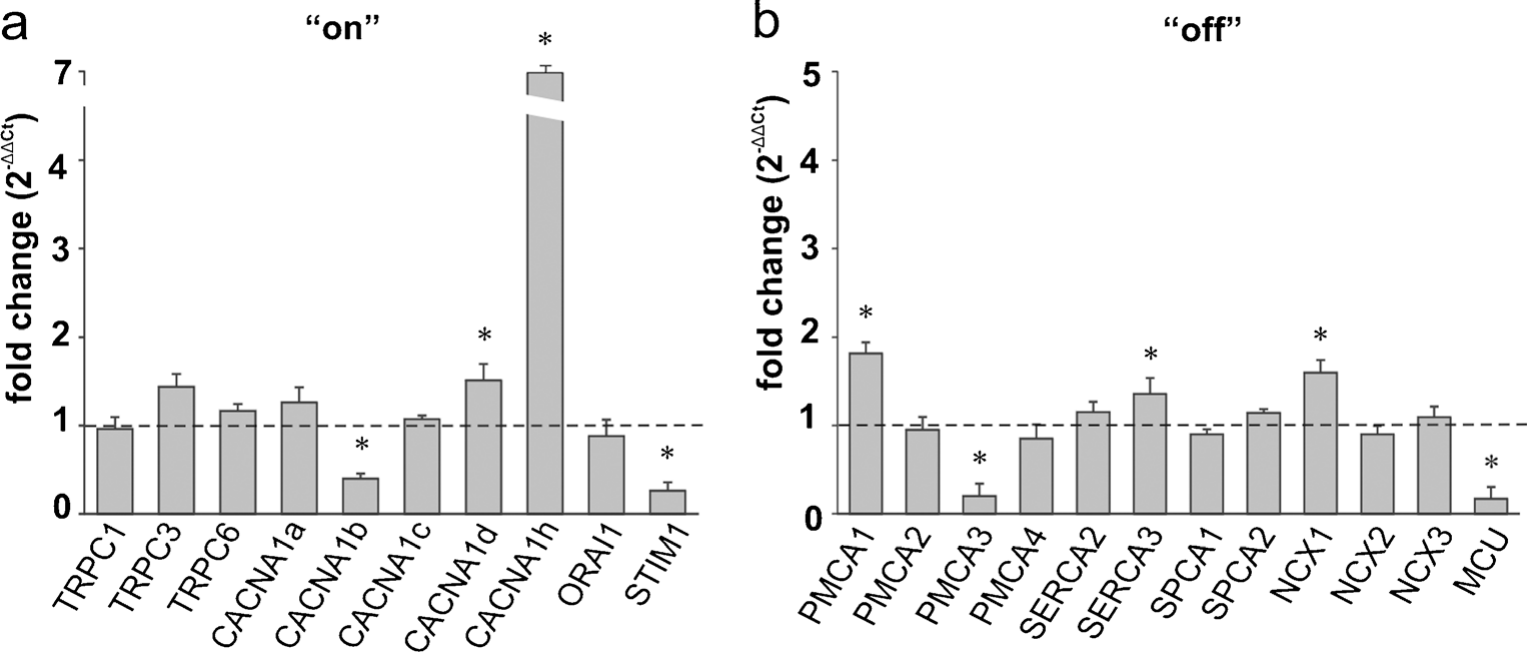
Ketamine-induced changes in the expression of the participants in the Ca^2+^-handling system in the cerebellum. The gene expression level for the “on” (**a**) and “off” (**b**) systems was assessed by real-time PCR. The results are presented as relative units obtained after normalization to the Gapdh reference gene. The expression level of the target gene in control samples (saline-injected rats) was taken as 1 (*dotted line*). Statistical differences between saline-treated and ketamine-treated rats are indicated, *P < 0.05. *n* = 12

The most prominent up-regulation within the “on” systems was detected for CACNA1h (*P* = 0.0075) and a slight but statistically significant increase was seen for CACNA1d (*P* = 0.013), whereas ketamine administration caused a decline in the CACNA1b mRNA level (*P* = 0.0069) by ∼60 % (Fig. 6a). A strong reduction of STIM expression level was observed (*P* = 0.0046). Moreover, the cerebellum “off” systems showed an enhanced expression of PMCA1 (*P* = 0.040), SERCA3 (*P* = 0.015), and NCX1 (*P* = 0.045) in response to ketamine but a reduction of nearly 80 % in mRNA level for PMCA3 (*P* = 0.047) and MCU (*P* = 0.035) occurred (Fig. 6b).

#### Hippocampus

**Fig. 7:**
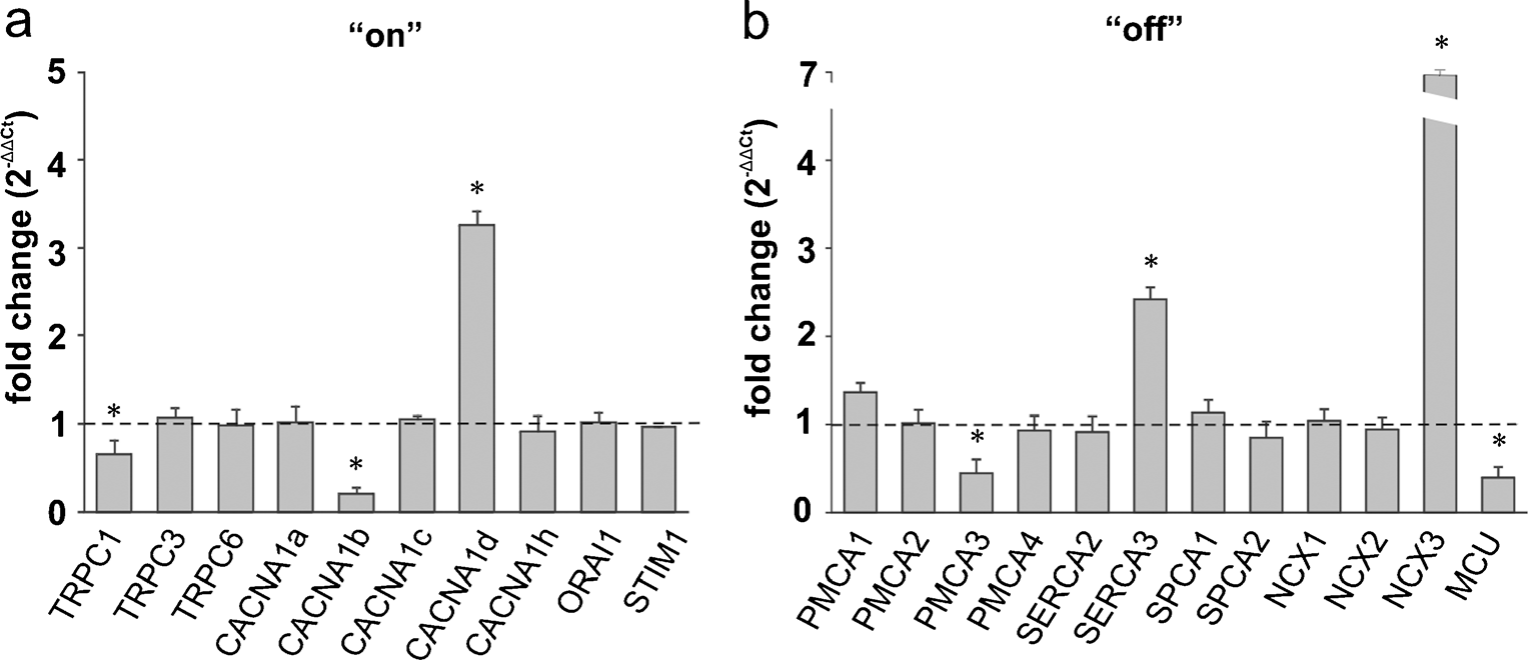
Ketamine-induced changes in the expression of the participants in the Ca^2+^-handling system in the hippocampus. The gene expression level for the “on” (**a**) and “off” (**b**) systems was assessed by real-time PCR. The results are presented as relative units obtained after normalization to the Gapdh reference gene. The expression level of the target gene in control samples (saline-injected rats) was taken as 1 (*dotted line*). Statistical differences between saline-treated and ketamine-treated rats are indicated, *P < 0.05. *n* = 12

Pronounced changes in mRNA level were observed for both calcium “on” and “off” systems in the hippocampus (Fig. 7a, b). Administration of the drug tended to decrease the expression of CACNA1b (*P* = 0.038), TRPC1 (*P* = 0.043), PMCA3 (*P* = 0.039) and MCU (*P* = 0.018), the last two actively participating in Ca^2+^ clearance. The greatest increase in response to ketamine was found for NCX3 mRNA (*P* = 0.013; Fig. 7b). In addition, ketamine up-regulated CACNA1d (*P* = 0.015) and SERCA3 (*P* = 0.038).

#### Striatum

**Fig. 8:**
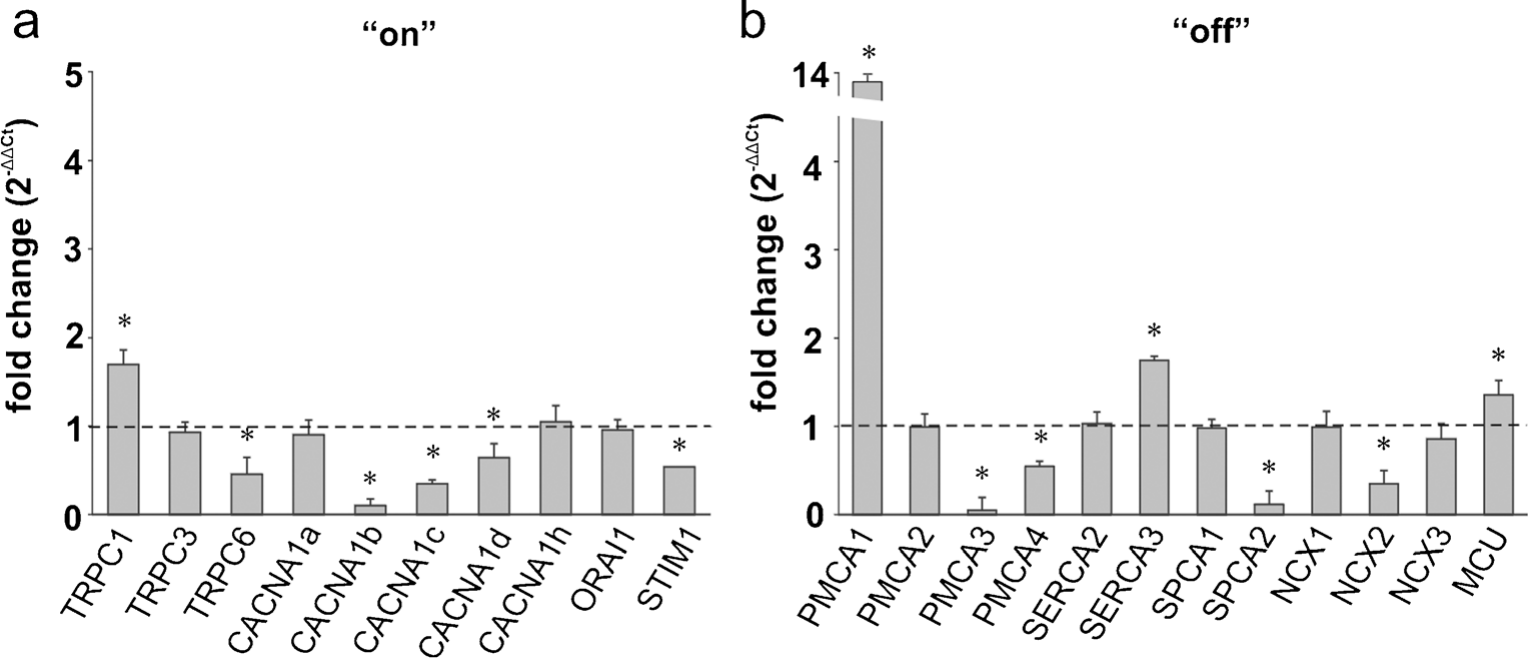
Ketamine-induced changes in the expression of the participants in the Ca^2+^-handling system in the striatum. The gene expression level for the “on” (**a**) and “off” (**b**) systems was assessed by real-time PCR. The results are presented as relative units obtained after normalization to the Gapdh reference gene. The expression level of the target gene in control samples (saline-injected rats) was taken as 1 (*dotted line*). Statistical differences between saline-treated and ketamine-treated rats are indicated, *P < 0.05. *n* = 12

In the striatum, ketamine induced the most intensive changes in the expression pattern of the Ca^2+^ homeostasis participants (Fig. 8a). This was manifested by the altered expression of 13 out of 22 examined genes. The expression of genes encoding calcium “on” elements were mostly down-regulated. Indeed, we detected lowered mRNA levels for TRPC6 (*P* = 0.0038), CACNA1b (*P* = 0.0082), CACNA1c (*P* = 0.016) and CACNA1d (*P* = 0.040). The only exception was TRPC1, whose expression increased by nearly 70 % (*P* = 0.040). The expression of a member of the store-operated Ca^2+^ entry mechanism, namely STIM1, was reduced (*P* = 0.047). Some of the most pronounced changes ascribed to ketamine treatment were found in the calcium “off” systems including a substantial up-regulation of PMCA1 (*P* = 0.042), MCU (*P* = 0.032), and SERCA3 (*P* = 0.043) genes, but a decline in PMCA3 (*P* = 0.0004), PMCA4 (*P* = 0.026), SPCA2 (*P* = 0.038) and NCX2 (*P* = 0.028) mRNA level was detected (Fig. 8b).

### Analysis of correlation between Ca^2+^-handling system transcripts level and intracellular [Ca^2+^]_c_

**Fig. 9:**
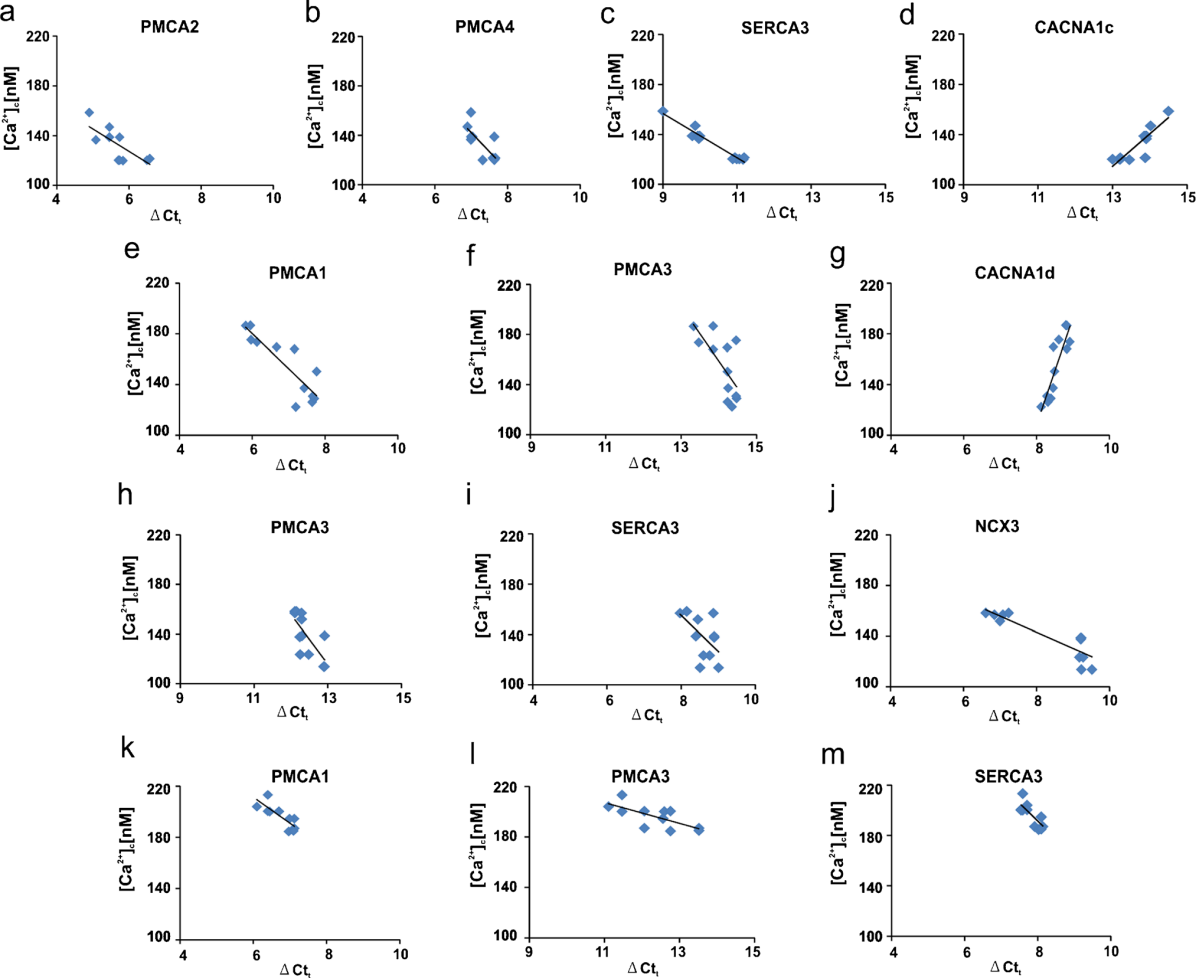
Correlations between expression of the participants of the calcium-handling systems and [Ca^2+^]_c_ in ketamine-treated rats. The graphs present the basal calcium concentration plotted as a function of the expression level for each gene. Only relevant correlations are shown. The *line* on the graph represents the regressive line. **a–d** Cortex. **e–g** Cerebellum. **h–j** Hippocampus. **k–m** Striatum. *n* = 12

To verify the potential relationship between increased [Ca^2+^]_c_ and the changes in the expression of the members of the calcium-handling systems, we performed a correlation analysis. A significant negative correlation was revealed in the cortex for PMCA2 (*r* = −0.64, *P* = 0.047; Fig. 9a), PMCA4 (*r* = −0.73, *P* = 0.023; Fig. 9b) and SERCA3 (*r* = −0.96, *P* = 0.036; Fig. 9c) and a positive correlation was observed for CACNA1c (*r* = 0.77, *P* = 0.04; Fig. 9d). In the cerebellum, a negative correlation was found for PMCA1 (*r* = −0.65, *P* = 0.043; Fig. 9e), PMCA3 (*r* = −0.71, *P* = 0.048; Fig. 9f) and CACNA1d (*r* = 0.80, *P* = 0.049; Fig. 9g). Moreover, significant negative correlations were detected for PMCA3 (*r* = −0.75, *P* = 0.032; Fig. 9h), SERCA3 (*r* = −0.72, *P* = 0.017; Fig. 9i) and NCX3 (*r* = −0.88, *p* = 0.031; Fig. 9j) in the hippocampus. Finally, striatal transcript levels showed a negative correlation with [Ca^2+^]_c_ only for PMCA1 (*r* = −0.66, *P* = 0.034; Fig. 9k), PMCA3 (*r* = −0.75, *P* = 0.016; Fig. 9l) and SERCA3 (*r* = −0.78, *P* = 0.026; Fig. 9m).

## Discussion

Assuming that schizophrenia is a disease of calcium signaling, the essential question to be asked is whether the up-regulation or down-regulation of Ca^2+^-dependent cellular transmission reveals a central molecular pathology. By using ketamine to mimic schizophrenic symptoms in rats, we have shown increased [Ca^2+^]_c_ in all of the examined brain regions; however, an altered cytosolic Ca^2+^ level is correlated with hyperlocomotor activity only for the cortex and striatum.

The phenomenon of calcium homeostasis dysregulation can be partially deduced from the ketamine-induced model of schizophrenia. Namely, a reduction of NMDAR drive and a concomitant decrease in Ca^2+^ flow result in the compensatory down-regulation of Ca^2+^ sequestering proteins (Lidow 2003). Blockade of NMDAR on gamma amino-butyric acid (GABA)-ergic interneurons attenuates GABA release and abolishes the inhibition of major excitatory pathways. One of the possible consequences is a stimulation of Ca^2+^ entry through glutamate-independent pathways and Ca^2+^ mobilization from internal stores (Lidow 2003). Therefore, the overall effect of NMDAR blockade is a prolongation of calcium dynamics and a [Ca^2+^]_c_ increase in a large population of neurons. This increase can, in turn, inhibit NMDAR causing the dopamine D2 receptor-dependent potentiation of the ecytosolic calcium signal. The dopamine system is known to interact closely with Ca^2+^ signaling and both phasic dopamine release and the activity of D2 receptors are dependent on the Ca^2+^ influx or Ca^2+^ release from internal stores (Lidow 2003). The connection between an abnormally elevated Ca^2+^ level and schizophrenia-like symptoms suggested in our study is additionally highlighted by recent findings showing the ketamine-induced potentiation of dopamine release and the reduction of GABA secretion from rat striatum (Grasshoff et al. 2005; Meyer and Louilot 2012). This finding agrees with the original dopamine hyperactivity hypothesis of schizophrenia, which is further supported by several studies showing the increased density of the striatal dopamine D2 receptor (Lajtha et al. 2009; Lidow 2003).

Based on the well-established model of ketamine-induced experimental psychoses, we demonstrated a correlation between altered calcium signaling in the cortex and striatum and the generation of behavioral abnormalities. The prefrontal cortex stands out as one of the main regions affected in schizophrenia and the postnatal inactivation of this structure in rats promotes behavioral alterations similar to ketamine-evoked psychotic symptoms (Usun et al. 2013). Usun et al. (2013) also found a ketamine-induced potentiation of striatal dopamine release in adult rats following the postnatal blockade of the prefrontal cortex. Dysfunctional interaction between cortical and striatal dopamine systems is strongly suggested to underlie the impaired motor function observed in our study. In agreement with the above, we propose that ketamine alters neural Ca^2+^ homeostasis by blocking NMDAR located on GABAergic neurons in the striatum and prefrontal cortex; this might influence Ca^2+^-dependent dopamine release resulting in increased neural activity. Among GABAergic neurons, prefrontal cortical parvalbumin interneurons seem to be highly sensitive to NMDA antagonists. Chronic exposure to ketamine leads to a reduction of GAD67 and parvalbumin expression in parallel with the activation of NADPH oxidase and superoxide accumulation. Blocking of NADPH oxidase prevented these actions, a finding that indicates this enzyme as a source of parvalbumin interneuron vulnerability. Therefore, psychotomimetic effects generated by NMDAR antagonists can be mediated, at least in part, by oxidative stress produced by NADPH oxidase (Behrens et al. 2007; Zhang et al. 2008). Additionally, chronic stress and subsequent dysregulation of calcium homeostasis have been shown to underlie the loss of parvalbumin-immunoreactive cells, an event that is thought to impair GABAergic network function. The physiological consequence of this deficit is the altered rhythmic oscillations commonly observed in schizophrenic patients (Hu et al. 2010; Filipović et al. 2013).

So far, most of the reports have focused on the relationship between defective postsynaptic glutamatergic neurotransmission and schizophrenia-related symptoms. However, a growing body of evidence has also revealed presynaptic glutamatergic alterations, particularly connected with proteins involved in synaptic vesicles fusion and exocytosis (Oni-Orisan et al. 2008; Granseth et al. 2015). These reports strongly indicate that the expression of vesicular glutamate transporters VGLUT1 and 2 is changed in schizophrenia. Because VGLUTs contribute to the machinery that facilitates glutamate release, the down-regulation of transporter expression is expected to alter glutamate neurotransmission and synaptic activity; indeed, gene-specific decreases in VGLUT mRNA have been suggested in schizophrenia (Eastwood and Harrison 2005). The importance of VGLUTs is further supported by studies with full knock-out mouse models. VGLUT1 full knock-out mice die after weaning, as do VGLUT2-deficient animals, which die immediately after birth. Interestingly, mice with conditional VGLUT knock-out showed hyperactivity, impaired social behaviors and cognitive deficiency and displayed prepulse inhibition and, therefore, can be considered as a hypoglutamate model of schizophrenia-like symptoms (Wallén-Mackenzie et al. 2010; Inta et al. 2012).

Also of interest, among the analyzed genes encoding calcium-handling factors, we found only a few whose expression is correlated with increased [Ca^2+^]_c_. In order to avoid an exaggerated analysis, we decided to limit our discussion of the potential effect of ketamine on brain calcium homeostasis only to these genes. In the cerebellum, hippocampus and striatum, ketamine administration leads to a down-regulation of PMCA3. This PMCA isoform and PMCA2, whose expression is reduced in the cortex, are the fastest PMCA isoforms and their presence is thought to sensitize neuronal cells even to subtle changes in [Ca^2+^]_c_. Although the data regarding specific PMCA3 function are scarce, several studies of neuronal cultures have shown that the reduced expression of either PMCA2 or PMCA3 decreases Ca^2+^-clearing potency making cells unable to maintain Ca^2+^ homeostasis (Fernandes et al. 2007; Boczek et al. 2012). A slower return of Ca^2+^ transient to the baseline and the compromised ability to oppose the influx of calcium through the plasma membrane capacitive channels have recently been attributed to dysfunctional PMCA3 (Zanni et al. 2012). In addition to affecting calcium clearance, decreased levels of PMCA2 have also been associated with neuronal pathology and a reduced number of motor neurons has been revealed in PMCA2-deficient mice (Kurnellas et al. 2005). Therefore, the lowered expression of PMCA2 or PMCA3 observed in our study might form the basis of the inability to maintain calcium homeostasis following ketamine administration. This, in turn, might contribute to the ketamine-induced neuronal death observed in primates or even to the neuronal loss reported in schizophrenic patients (Lajtha et al. 2009).

Increased expression of SERCA3 in the cortex, hippocampus and striatum was correlated with a higher [Ca^2+^]_c_ level in these brain structures, suggesting a compensatory change by sequestration of calcium to intracellular compartments. However, the changes in the Ca^2+^ content in the internal stores depend upon the balance between the activity of SERCA pumps and Ca^2+^ leakage or release back into the cytosol. A massive calcium efflux from the ER is not the reason for the increased [Ca^2+^]_c_ in ketamine-treated brains observed in our study, as ketamine has been shown to decrease the release of internally stored Ca^2+^ (Xiang et al. 2008). Therefore, the higher SERCA3 expression might indicate the more efficient packing of Ca^2+^ into the lumen of the ER aimed at reducing the abnormally high cytosolic Ca^2+^ level. A similar reason can be suggested for the up-regulation of PMCA1 in the striatum and cerebellum, PMCA4 in the cortex and NCX3 in the hippocampus. The beneficial role of PMCA and SERCA overexpression on the reduction and increase in the ER [Ca^2+^] level, respectively, has previously been demonstrated in vivo (Brini et al. 2000). Additionally, the compensatory up-regulation of PMCA4 has been revealed in the anterior temporal lobes in schizophrenic patients (Martins-de-Souza et al. 2009).

Calcium signaling through CACNA1 has been implicated in several brain disorders and the CACNA1c variant has been associated with schizophrenia (Lajtha et al. 2009). This has been confirmed by recent genome-wide association studies showing CACNA1c as a susceptibility gene for schizophrenia in the worldwide population (Hamshere et al. 2013; Ripke et al. 2013; Zheng et al. 2014). Despite these findings, data regarding the clinical effectiveness of calcium channel blockers in schizophrenia treatment are scarce. So far, several reports have only demonstrated the efficient use of verapamil, diltiazem, nifedipine and nimodipine as an adjunct therapy, alleviating side effects such as tardive dyskinesia caused by neuroleptic drugs (Hollister and Trevino 1999; Essali et al. 2011). In our study, an increased expression of subunits 1c and 1d (L-type VDDCs) was observed in the cortex and cerebellum, respectively but this might have a rather minor contribution to the overall dysregulation of calcium homeostasis, possibly because ketamine has been shown to inhibit the L-type dependent Ca^2+^ current (Yamakage et al. 1995). Instead, we assume that it might affect the proper function of numerous neurological circuits, since L-type voltage-dependent Ca^2+^ channels have been found to be engaged in mesolimbic-mediated behaviors and in the hippocampal formation of spatial memory in rodents (Bhat et al. 2012).

In conclusion, we have shown that the inability to maintain brain Ca^2+^ homeostasis in ketamine-treated rats might be caused by an imbalanced expression between the calcium “on” and “off” mechanisms. The key changes correlating with higher [Ca^2+^]_c_ involve the dysfunctional expression of the calcium extruding systems, with a prevalence of PMCA2 and PMCA3. Although various rescue mechanisms, i.e., the increased expression of constitutive PMCA isoforms or SERCA pumps, are activated in discrete brain regions, a finding possibly related to their functional specificity, they are still inefficient with regard to the restoration of the balance of calcium ions. Consecutively, an abnormal calcium signal can be linked with behavioral abnormalities; such a correlation has been revealed here for the cortex and striatum. The identification of genes whose expression is affected by ketamine treatment, in conjunction with the changes reported in schizophrenia, indicate their involvement as putative etiological factors of this disease.
